# Lymphocyte recovery from radiation-induced lymphopenia in locally advanced esophageal squamous cell carcinoma: correlations with prognosis and lymphocyte-related organs

**DOI:** 10.1186/s13014-023-02354-w

**Published:** 2023-10-19

**Authors:** Ihsuan Tseng, Dashan Ai, Yun Chen, Hongcheng Zhu, Fangfang Li, Yang Xu, Lu Yu, Qi Liu, Jiaying Deng, Shengnan Hao, Zhengfei Zhu, Weixin Zhao, Min Fan, Ling Li, Fengtao Su, Kuaile Zhao

**Affiliations:** 1 Department of Radiation Oncology, Fudan University Shanghai Cancer Center, Shanghai, 200032 China; 2 Department of Oncology, Shanghai Medical College, Fudan University, Shanghai, 200032 China; 3 Shanghai Clinical Research Center for Radiation Oncology, Shanghai, 200032 China; 4 Shanghai Key Laboratory of Radiation Oncology, Shanghai, 200032 China; 5 Center for Cancer Immunology, Institute of Biomedicine and Biotechnology, Shenzhen Institute of Advanced Technology, Chinese Academy of Sciences, Shenzhen, 518055 China; 6 Department of Medicine, Enhance Human Health Through Pharma Technology Innovation, Shanghai, 201800 China; 7 Shanghai Medical College of Fudan University, Shanghai, 200032 China; 8 Cancer Institute, Fudan University Shanghai Cancer Center, Shanghai Medical College of Fudan University, Shanghai, 200032 China

**Keywords:** Esophageal cancer, Chemoradiotherapy, Lymphocyte, Recovery

## Abstract

**Background:**

Limited studies explored the relationship between lymphocyte recovery after definitive concurrent chemoradiotherapy (dCCRT) and prognosis in esophageal squamous cell carcinoma (ESCC).

**Methods:**

ESCC patients with obtainable absolute lymphocyte counts (ALCs) at 6 months after dCCRT were screened from prospective trials. Patients were divided into groups according to the grade of ALC nadir during radiotherapy (G4 or G1–3) and lymphocyte recovery status, which was assessed by lymphocyte recovery index (LRI), calculated as the ratio of post- to pre-treatment lymphocyte counts. Cox analysis was conducted to evaluate the prognostic significance of lymphocyte recovery status. Irradiated relative volumes of the bone marrow (BM) and spleen and effective dose to immune cells (EDIC) were collected to identify their impacts on lymphocyte recovery status by logistic analysis.

**Results:**

232 patients were enrolled. In 69 patients with G4 ALC nadir (group A and B) and 163 patients with G1–3 ALC nadir (group C and D) during dCCRT, 27 (group A) and 67 (group C) patients showed an insufficient level of lymphocyte recovery (LRI < 60%), and 42 (group B) and 96 (group D) patients showed a satisfactory level of lymphocyte recovery (LRI ≥ 60%). Cox multivariable analysis revealed that inadequate lymphocyte recovery was significantly associated with worse overall survival (HR, 2.80 and 1.70) and local recurrence-free survival (HR, 2.82 and 1.60) both in group A vs group B and group C vs group D. Logistic analysis identified BM V5 (OR 4.24 and 2.29) as an independent predictor of inadequate lymphocyte recovery from G4 or G1–3 ALC nadir, respectively.

**Conclusions:**

Insufficient lymphocyte recovery might serve as a valuable prognostic factor, regardless of whether patients experienced G4 or G1–3 ALC nadir during radiotherapy. Additionally, it was observed that a larger relative volume of BM receiving ≥ 5 Gy was correlated with a higher risk of insufficient lymphocyte recovery.

**Supplementary Information:**

The online version contains supplementary material available at 10.1186/s13014-023-02354-w.

## Introduction

Radiotherapy as a recognized anti-tumor approach, is a double-edged sword as it also leads to the depletion of immune cells [1, 2]. Lymphocytes as vital immune cells in response to immunotherapy [3], are quite radiosensitive, being easily eliminated under exposure to as little as 2 Gy [4]. The clinical significance of severe lymphopenia during radiotherapy has already been evidenced by inferior survival outcomes in many solid tumors [2]. With the emergence of immunotherapy as a promising anti-cancer treatment, the significance of lymphocyte recovery from lymphopenia has gained increasing attention.

The recovery of lymphocyte counts after radiotherapy is a time-consuming process [5]. A study conducted in pancreatic cancer showed a clear correlation between lymphocyte recovered within 6 months of initiating chemoradiotherapy (CRT) and better clinical outcomes, and indicated lymphocyte counts at baseline and planning target volume (PTV) as independent factors related to lymphocyte recovery [6]. Whereas, the relevance of lymphocyte recovery at 6–8 weeks after CRT to long-term outcomes appeared to be disconnected in esophageal cancer [7].

Building upon previous research findings, this study aimed to explore the relationship between lymphocyte recovery from radiation-induced lymphopenia and survival outcomes in patients with esophageal squamous cell carcinoma (ESCC) who underwent definitive concurrent chemoradiotherapy (dCCRT), and further to figure out which specific lymphocyte-related organ at risk exhibit a stronger correlation with lymphocyte recovery.

## Methods

### Patient selection

ESCC patients treated with dCCRT were screened from two prospective randomized clinical trials known as ESO Shanghai 1 (*NCT01591135*) [8] and ESO Shanghai 2 (*NCT02459457*) [9]. All patients were scheduled to receive a total dose of 61.2 Gy delivered in 34 fractions (5 days/week, 1.8–2.0 Gy/d), using intensity-modulated radiation therapy (IMRT) with involved-field irradiation (IFI). Four chemotherapy regimens were included as follows: (1) PF: fluorouracil (5-FU) with cisplatin (DDP); TF: 5-FU with paclitaxel (PTX); (3) TP: PTX with DDP; (4) TC: PTX with carboplatin (CBP). Based on the screened population in our previous study [10], we next selected patients with accessible ALC data at 6 months (± 1 month) after the completion of dCCRT, which were collected before any extra administration of anti-tumor treatment due to disease progression (Additional file 1: Figure S1). Ethical review and approval were obtained from the appropriate ethics committee, and informed consent was completed directly by each patient [8, 9].

### Follow-up

The patients were followed up every 3 months for the first two years after the whole treatment and then every 6 months to the fifth year, when the follow-up time could be prolonged to every year in no exceptional circumstances. The follow-up duration lasted for at least 6 years. Overall survival (OS), progression-free survival (PFS), local recurrence-free survival (LRFS) and distant metastasis-free survival (DMFS) were recorded [8, 9].

### Data of absolute lymphocyte counts

Absolute lymphocyte counts (ALCs) were collected before dCCRT (at baseline), each week during dCCRT, and 6 (± 1) months after dCCRT. Based on CTCAE version 5.0 and the lower normal limit of ALC in our hospital (1.1 × 10^9^/L), lymphopenia is defined as ALC < 1.1 × 10^9^/L; the degree of lymphopenia is divided into 0–4 levels as follows: 0 (≥ 1.1 × 10^9^/L); 1 (< 1.1–0.8 × 10^9^/L), 2 (< 0.8–0.5 × 10^9^/L), 3 (< 0.5–0.2 × 10^9^/L) and 4 (< 0.2 × 10^9^/L). The lowest ALCs during dCCRT were identified by G0–4. The status of lymphocyte recovery was estimated by lymphocyte recovery index (LRI), which was defined as the ratio of ALC at 6 months after the end of chemoradiotherapy and ALC at baseline.

$$ {\text{lymphocyte recovery index (LRI)}} = \frac{{{\text{ALC at }}\;{6 }\;{\text{months after dCCRT}}}}{{{\text{ALC at baseline}}}}{*}100{{\% }} $$

### Patient grouping

The optimal cut-point value for LRI was determined by maximally selected log-rank statistics based on OS. Patients with insufficient lymphocyte recovery (LRI < cut-off) were regarded as “unrecovered”, while those with adequate lymphocyte recovery (LRI ≥ cut-off) were regarded as “recovered”. Then based on the grade of lymphocyte nadir during dCCRT (G4 and G1–3), the population were categorized into 4 groups: (1) Group A (G4 → unrecovered); (2) Group B (G4 → recovered); (3) Group C (G1–3 → unrecovered); (4) Group D (G1–3 → recovered).

### Dose-volume parameters

The body, heart, lungs, and spleen were outlined according to RTOG 1106 Atlas. The mean dose of heart, lungs and body were combined to calculate the effective dose to immune cells (EDIC) developed by Jin et al. [11] The delineation of the bone marrow (BM) and spleen were described in our previous research [10]. The mean doses and the relative volumes of BM and spleen receiving 5, 10, 20, 30, and 50 Gy (V5, V10, V20, V30, and V50) determined by dose-volume histogram (DVH) analysis.

### Statistical analysis

Continuous variables in clinical characteristics were categorized by median splits. The optimal cut-off point of LRI taken the integer portion was determined by the maximally selected log-rank statistics based on OS using the R package “maxstat” [12]. Kaplan Meier analysis and log-rank test were used to compare the differences in survival outcomes between groups. The hazard ratio (HR), 95%CI and corresponding *p*-value of each variable was calculated in the Cox model. Dose-volume parameters predicting lymphocyte recovery were transformed into binary variables by receiver operating characteristic curve (ROC) analysis and the organ-specific parameter with the lowest *p* value on univariate was chosen into multivariable adjustment. Univariable and multivariable logistic regression analysis were performed to correlate lymphocyte recovery with dose-volume parameters. Variables with *p* < 0.1 on univariable Cox/Logistic analysis were input for the following multivariable analysis. A two-tailed *p* < 0.05 was considered statistical significance. R studio version 4.2.1 (The R Foundation for Statistical Computing, Vienna, Austria) and Graphpad Prism version 9.0 (GraphPad Software Inc., San Diego, CA, USA) were applied in this study.

## Results

### Patient characteristics

Two hundred thirty-two eligible patients were enrolled for this study, including 185 (79.7%) males and 47 (20.3%) females. The median age of all patients was 62 years. Most of them (69.8%) were in normal health conditions with ECOG-PS-0. Fifty-eight (25.0%) patients at stage II and 137 (59.1%) patients with tumors at cervical and upper esophagus. The median tumor length was 5.0 cm. Of 232 patients, 94.8% finished the 61.2 Gy radiotherapy course. The number of patients treated with PF, TF, TP and TC was 64 (27.6%), 102 (44.0%), 30 (12.9%) and 36 (15.5%), respectively. After concurrent chemoradiotherapy, 178 (76.7%) patients received 2 cycles of consolidation chemotherapy and 54 (23.2%) patients received 0–1 cycle. Clinical characteristics in details were listed in Table 1.

**Table 1:** Patient characteristics

	Total (n = 232, %)
Gender	
Male	185 (79.7)
Female	47 (20.3)
Age	
≤ 62 years	127 (54.7)
> 62 years	105 (45.3)
ECOG-PS	
0	162 (69.8)
1–2	70 (30.2)
Tumor stage*	
II	58 (25.0)
III + IV	174 (75.0)
Tumor location	
Cervical + upper	137 (59.1)
Middle + lower + multiple	95 (40.9)
Length	
≤ 5.0 cm	118 (50.9)
> 5.0 cm	114 (49.1)
Radiotherapy dose	
61.2 Gy	220 (94.8)
50.4 ~ < 61.2 Gy	12 (5.2)
Chemo regimen	
PF	64 (27.6)
TF	102 (44.0)
TP	30 (12.9)
TC	36 (15.5)
Concurrent chemo completion	
Yes	197 (84.9)
No	35 (15.1)
Consolidation chemo cycles	
2 cycles	178 (76.7)
0–1 cycle	54 (23.2)

ECOG-PS: Eastern Cooperative Oncology Group performance status; PF: fluorouracil (5-FU) with cisplatin (DDP); TC: PTX with carboplatin (CBP); TF: 5-FU with paclitaxel (PTX); TP: PTX with DDP

*According to AJCC 6th

### ALC data and patient subgroups

**Fig. 1:**
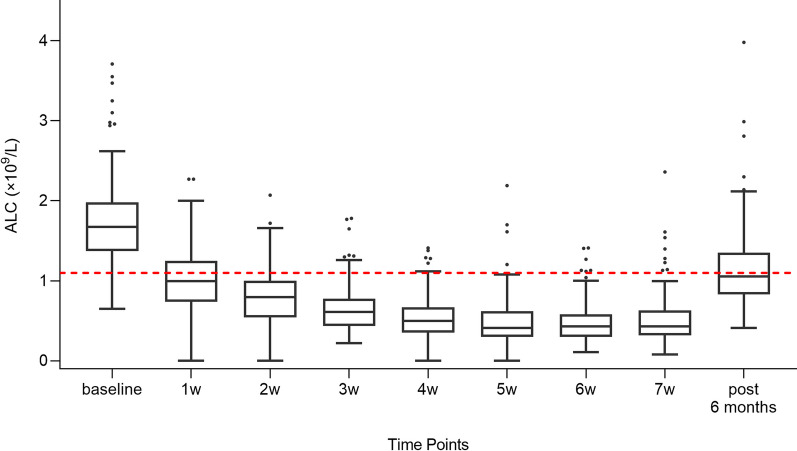
The dynamic changes of ALCs from baseline (before dCCRT) through week 1–7 during dCCRT and 6 months after the end of dCCRT represented by Tukey box-and-whisker plots. The red dashed line represented the lower limit of normal lymphocyte counts (1.1 × 10^9^/L). *ALC* absolute lymphocyte count, *dCCRT* definitive concurrent chemoradiotherapy

The ALC data of 232 patients were obtained. During treatment, ALCs declined every week and generally reached a plateau at week 5, continuing till the end of treatment, then gradually elevated to near-normal levels (Fig. 1). The median of ALCs at baseline was (× 10^9^/L) 1.68, and 0.99, 0.80, 0.61, 0.50, 0.41, 0.43, 0.43 for week1–7 during dCCRT, respectively. The cumulative incidence of G4 ALC nadir and G1–3 nadir was 29.7% (N = 69), and 70.3% (N = 163).

Six months after the end of dCCRT, there were 104 (44.8%) patients whose ALCs returned to normal level (≥ 1.1 × 10^9^/L), while more than half of them (55.2%) were still accompanied by varying degrees of lymphopenia: 77 (33.2%) with grade 1 lymphopenia, 47 (20.3%) with grade 2 and 4 (1.7%) with grade 3. The median of ALC at 6 months after dCCRT was 1.06 × 10^9^/L (range 0.41–3.98).

With regard to the status of lymphocyte recovery the median of LRI was 67.8% (range 19.8–193.1). According to the cut-off point of LRI (60%) by maxstat based on OS (Additional file 1: Figure S2) and the development of G4 and G1–3 ALC nadir during dCCRT, the population were classified into 4 groups: group A (G4 → unrecovered) included patients with G4 ALC nadir during dCCRT and LRI < 60% (N = 27, 11.6%), group B (G4 → recovered) with G4 ALC nadir during dCCRT and LRI ≥ 60% (N = 42, 18.1%). For patients with G1–3 ALC nadir during dCCRT, group C (G1–3 → unrecovered) included those with LRI < 60% (N = 67, 28.9%), while group D (G1–3 → recovered) with LRI ≥ 60% (N = 96, 41.4%). Distributions of clinical characteristics in these 4 groups could be found in Additional file 2: Table S1.

### Prognosis of lymphocyte recovery combined with ALC nadir during dCCRT

**Fig. 2:**
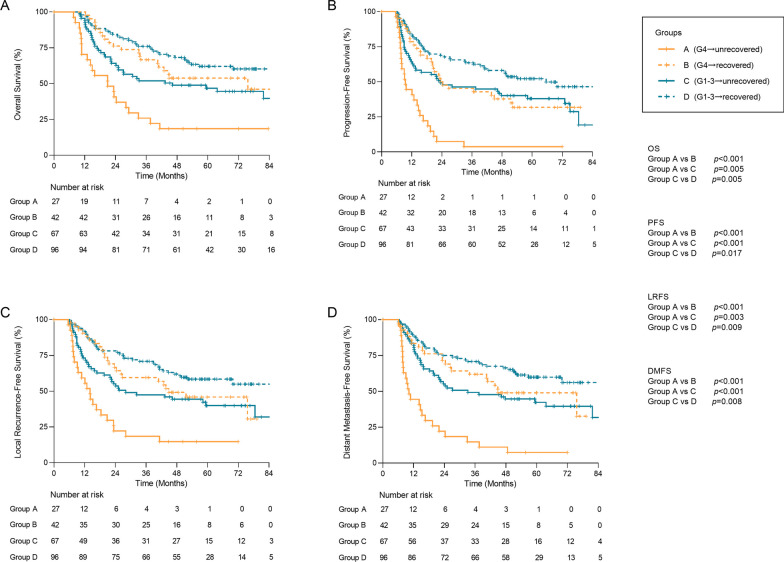
Kaplan Meier curves of **A** overall survival, **B** progression-free survival; **C** local recurrence-free survival, and **D** distant metastasis-free survival between 4 groups. Group A (G4 → unrecovered) included patients with G4 ALC nadir during dCCRT and LRI < 60% (N = 26), group B (G4 → recovered) with G4 ALC nadir during dCCRT and LRI ≥ 60% (N = 42). For patients with G1–3 ALC nadir during dCCRT, group C (G1–3 → unrecovered) included those with LRI < 60% (N = 67), while group D (G1–3 → recovered) with LRI ≥ 60% (N = 96). ALC, absolute lymphocyte count dCCRT, definitive concurrent chemoradiotherapy; DMFS, distant metastasis-free survival; LRFS, local recurrence-free survival; LRI, lymphocyte recovery index; OS, overall survival; PFS, progression-free survival

At analysis, 117 (50.4%) patients died with 69.9 months of median OS time. The overall PFS, LRFS and DMFS median time was 27.4 months, 46.2 months and 49.2 months, respectively. As displayed in Fig. 2, among patients with G4 ALC nadir during dCCRT, significantly poorer 5-year OS rate (18.5% vs 53.8% *p* < 0.001) and 5-year PFS rate (3.7% vs 31.7%, *p* < 0.001) were observed in group A vs group B. Also, prominent differences in the 5-year LRFS rate (14.8% vs 45.9%, *p* < 0.001) and 5-year DMFS rate (7.4% vs 49.0%, *p* < 0.001) existed in group A vs group B. Among patients with G1–3 ALC nadir during dCCRT, survival curve analysis indicated poorer 5-year OS (46.8% vs 62.1%, *p* = 0.005) and 5-year PFS (38.0% vs 52.2%, *p* = 0.017) in group C vs group D. The 5-year LRFS rate (58.4% vs 39.9%, *p* = 0.009) and 5-year DMFS rate (59.9% vs 42.3%, *p* = 0.008) in group D was respectively significantly higher than that in group C.

Comparing group A with group C, OS and PFS were both worse in patients with G4 ALC nadir than those with G1–3 ALC nadir, with a 5-year OS rate 18.5% vs 46.8% months (*p* = 0.005) and 5-year PFS rate 3.7% vs 38.0% (*p* < 0.001), respectively. Besides, poorer 5-year LRFS rate (14.8% vs 39.9%, *p* = 0.003) and 5-year DMFS rate (7.4% vs 42.3%, *p* < 0.001) were observed in group A vs group C.

Based on univariable Cox analysis (Additional file 2: Tables S2, S3), following multi-factor adjustment in Table 2 revealed that lymphocyte unrecovered from G4 ALC nadir at post 6 months was independently related to shorter OS (HR, 2.80; 95% CI 1.47–5.34; *p* = 0.002) and PFS (HR, 3.67; 95% CI 2.09–6.46; *p* < 0.001), as well as poorer LRFS (HR, 2.82; 95% CI 1.51–5.26; *p* = 0.001) and DMFS (HR, 3.65; 95% CI 1.96–6.78; *p* < 0.001). Among patients with G1–3 ALC nadir during dCCRT, inadequate lymphocyte recovery was still found to be an independent factor associated with poorer OS (HR, 1.70; 95% CI 1.07–2.72; *p* = 0.025) and LRFS (HR, 1.60; 95% CI 1.01–2.52; *p* = 0.040). However, no statistically prognostic significance was observed in PFS and DMFS (Additional file 2: Table S4, Fig. 2).

**Table 2:** Multivariable Cox analysis for survival outcomes between group A and B

	OS		PFS		LRFS		DMFS	
	HR (95% Cl)	*p*	HR (95% Cl)	*p*	HR (95% Cl)	*p*	HR (95% Cl)	*p*
Tumor stage*		0.371	–	–		0.308		0.375
II	0.66 (0.27–1.64)				0.63 (0.25–1.54)		0.68 (0.30–1.58)	
III + IV	Ref				Ref		Ref	
Groups		**0.002**		** < 0.001**		**0.001**		** < 0.001**
Group A	2.80 (1.47–5.34)		3.67 (2.09–6.46)		2.82 (1.51–5.26)		3.65 (1.96–6.78)	
Group B	Ref		Ref		Ref		Ref	

ALC: absolute lymphocyte count; CI, confidence interval; DMFS, distant metastasis-free survival; ECOG-PS: Eastern Cooperative Oncology Group performance status; HR: hazard ratio; LRFS, local recurrence-free survival; LRI, lymphocyte recovery index; OS, overall survival; PF: fluorouracil (5-FU) with cisplatin (DDP); PFS, progression-free survival; TC: PTX with carboplatin (CBP); TF: 5-FU with paclitaxel (PTX); TP: PTX with DDP

*According to AJCC 6th. Group A included patients with G4 ALC nadir during dCCRT and LRI < 60% (N = 27) and group B with G4 ALC nadir during dCCRT and LRI ≥ 60% (N = 42)

### Prognosis of lymphocyte recovery within the same tumor stage

Based on the tumor stage, the population was re-stratified for further investigation. In the subset of patients with stage II (N = 58), no significant correlations were observed between lymphocyte recovery status and clinical outcomes (data not shown). However, within the stage III + IV group (N = 174, including 137 with stage III and 37 with stage IV), patients who did not experience sufficient lymphocyte recovery at 6 months post-therapy had inferior OS, PFS, LRFS and DMFS (Additional file 1: Figure S3, Additional file 2: Table S5). Furthermore, the multivariable Cox analysis in Additional file 2: Table S6 demonstrated an independent association between the inadequate lymphocyte recovery and shorter OS (HR, 1.77; 95% CI 1.19–2.64; *p* = 0.005), shorter PFS (HR, 1.64; 95% CI 1.15–2.35; *p* = 0.007), as well as inferior LRFS (HR, 1.65; 95% CI 1.13–2.42; *p* = 0.010) and inferior DMFS (HR, 1.80; 95% CI 1.23–2.65; *p* = 0.003).

### Dose-volume parameters

In 232 patients, the median of V5, V10, V20, V30 and V50 of the BM was 42.2% (range 12.6–69.2), 33.5% (range 9.3–54.2), 26.1% (range 5.5–41.8), 18.8% (range 3.3–34.7), 5.3% (range 0.5–16.2), respectively. The max of the spleen V5, V10, V20, V30 and V50 was 96.0%, 87.7%, 80.3%, 55.2% and 6.7%, respectively. For EDIC, the median dose was 9.0 Gy (range 2.0–14.6).

In group A vs B, the median of V5, V10, V20, V30 and V50 of the BM was 46.0% vs 40.7%, 37.2% vs 33.8%, 27.9% vs 25.7%, 20.3% vs 19.9% and 5.3% vs 5.4%, and the max values of spleen V5, V10, V20, V30 and V50 was 77.4% vs 96.0%, 60.0% vs 87.7%, 35.5% vs 80.3%, 7.9% vs 55.2% and 0 vs 1.4%, respectively. In group C vs D, the median of related BM dose-volume parameters mentioned above was respectively 44.7% vs 40.8%, 34.0% vs 32.2%, 26.3% vs 25.5%, 19.0% vs 17.6% and 5.5% vs 4.8%; and max values of spleen V5, V10, V20, V30 and V50 were 70.0% vs 87.0%, 60.5% vs 64.7%, 50.7% vs 53.8%, 32.4% vs 45.8% and 6.5% vs 6.7%. The median of EDIC in group A vs B and group C vs D was 14.1 Gy vs 13.6 Gy and 8.59 Gy vs 8.50 Gy, respectively.

### Predictors of lymphocyte recovery at 6 months after dCCRT

In univariable logistic regression analysis, tumor stage (II vs. III + IVa), dose volume parameters of BM gave *p*-values of less than 0.05 whether in comparison with group A and group B (Additional file 2: Table S7) or group C and group D (Additional file 2: Table S8). Between group A and group B, BM V5 ≥ 40.7% (OR 5.75; 95% CI 1.69–19.52; *p* = 0.005) was positively associated with lymphocyte unrecovery, while, BM V5 ≥ 46.0% (OR 2.91; 95% CI, 1.50–5.66; *p* = 0.002) for patients with G1–3 ALC nadir and lymphocyte unrecovery. Subsequently, multivariable adjustment indicated that BM V5 ≥ 40.7% (OR 4.24; 95% CI 1.18–15.20; *p* = 0.027) and ≥ 46.0% (OR 2.29; 95% CI 1.11–4.73; *p* = 0.025) was independently correlated with lymphocyte unrecovery 6 months after dCCRT respectively in patients with G4 and G1–3 ALC nadir (Table 3).

**Table 3:** Multivariable Logistic regression analysis of factors related to lymphocyte unrecovered from radiation-induced lymphopenia

	Group A versus Group B			Group C versus Group D	
	OR (95% Cl)	*p*		OR (95% Cl)	*p*
Tumor stage*		0.072	Tumor stage*		**0.023**
II	0.14 (0.02–1.20)		II	0.39 (0.17–0.88)	
III + IV	Ref		III + IV	Ref	
Consolidation chemo cycles		0.421	Consolidation chemo cycles	–	–
2 cycles	1.70 (0.47–6.16)		2 cycles		
0–1 cycle	Ref		0–1 cycle		
Bone marrow V5		**0.027**	Bone Marrow V5		**0.025**
≥ 40.7%	4.24 (1.18–15.20)		≥ 46.0%	2.29 (1.11–4.73)	
< 40.7%	Ref		< 46.0%	Ref	
EDIC	–	–	EDIC		0.460
≥ 11.8 Gy			≥ 10.3 Gy	1.32 (0.63–2.78)	
< 11.8 Gy			< 10. 3 Gy	Ref	

ALC: absolute lymphocyte count; dCCRT: definitive concurrent chemoradiotherapy; EDIC, the effective dose to immune cells; LRI, lymphocyte recovery index

*According to AJCC 6th. Group A included patients with G4 ALC nadir during dCCRT and LRI < 60% (N = 27); group B with G4 ALC nadir during dCCRT and LRI ≥ 60% (N = 42); group C included patients with G1–3 ALC nadir during dCCRT and LRI < 60% (N = 67) and group D with G1–3 ALC nadir during dCCRT and LRI ≥ 60% (N = 96)

## Discussion

To the best of our knowledge, this study represents the first exploration of lymphocyte recovery at 6 months after dCCRT in ESCC patients. We employed a novel index, integrating lymphocyte counts at the post-6-month mark with baseline counts, to demonstrate the status of lymphocyte recovery. The findings revealed that patients with inadequate lymphocyte recovery had poorer survival outcomes, simultaneously indicating the potential of this recovery index to differentiate prognosis in patients with G1–3 ALC nadir during dCCRT, which was previously overlooked. Furthermore, a multivariable analysis revealed a significant correlation between bone marrow irradiation and lymphocyte recovery.

It is widely acknowledged that circulating lymphocytes serve as promising markers for evaluating the systemic immune system [2].Notably, a study of pancreatic cancer conducted by Lee, et al. [6], revealed that patients who recovered from severe lymphopenia had better OS and PFS. Similarly, Cho, et al. [13], observed that lung cancer patients with persistent lymphopenia 3 months after CCRT had poorer OS and PFS. In contrast, Deng, et al. [7], reported that lymphocyte recovery at 6–8 weeks after CRT did not mitigate the negative impact on survival outcomes caused by radiation-induced G4 ALC nadir. Given the heterogeneity of lymphocyte recovery ability [14] and variations in immunologic status at baseline, we estimated the status of lymphocyte recovery by the calculating the ratio of ALCs at 6 months after dCCRT to ALCs at baseline. Then according to the degrees of severest lymphopenia during radiotherapy, patients were stratified into four groups for comparisons of survival outcomes and explorations of organs at risk related to lymphocyte recovery. Final results revealed that inadequate lymphocyte recovery at post-6 months was independently associated with poorer OS, PFS, LRFS and DMFS in patients with G4 ALC nadir. Even among patients with G1–3 ALC nadir, those without adequate lymphocyte recovery had inferior OS and LRFS. Separate analyses were conducted to evaluate the prognostic value of lymphocyte recovery status within the same stage, but significant correlations were not observed in the stage II group due to limited numbers. Similarly, due to the small sample size in the stage IV group, we merged these patients into the stage III group for analysis and found that lymphocyte recovery status could differentiate between favorable and unfavorable prognosis in the combined stage III + IV group.

Notably, Lee, et al. [6], found that a larger PTV was associated with impaired lymphocyte recovery in pancreatic cancer. On the other hand, Cho, et al. [13], failed to observe significant dose differences in large vessels between patients who experienced lymphocyte recovery and those who did not. In our study, we examined the correlation between lymphocyte recovery and the irradiation of the BM, spleen, and EDIC, which have been proven to do with the occurrence of G4 ALC nadir during radiotherapy [11, 15, 16]. Multivariable analysis showed that BM V5 was strongly correlated with lymphocyte recovery status both in the group of patients with G4 or G1–3 ALC nadir during dCCRT, respectively. The bone marrow, being an essential organ for lymphopoiesis, shows extremely sensitive to radiation, with a decrease of 50% volume in red BM observed at doses as low as 4 Gy in 1–2 weeks of radiotherapy initiation [17]. Via positron emission tomography (PET) examination, Noticewala, et al. [18] tested hematopoietic distributions between irradiated and non-irradiated bone marrow at baseline and within 1.5 to 6 months after CRT and then identified that higher mean pelvic bone marrow doses resulted in a weaker compensatory response in the medullary region. Demonstrable persistent effects on pelvic bone marrow were manifested in late lymphopenia at 12 months after radiotherapy in prostate cancer, showing more exposure irradiation to the pelvic marrow could end in feeble lymphocyte recovery [19]. Additionally, reduced lymphocyte counts within bone marrow were apparently observed in irradiated mice without tumor burden [20]. These shreds of evidence pointed out that higher irradiation dose to bone marrow is likely to damage medullary hematopoiesis and impair long-term recovery.

The weak correlations between lymphocyte recovery and other organs at risk might be attributed to the fact that blood vessels and the spleen serve as lymphocytic reserve organs rather than hemopoietic organs. Irradiation to circulating cells and the spleen was more likely to cause acute elimination of lymphocytes during radiotherapy instead of affecting lymphopoiesis. What cannot be ignored is that we used a surrogate to represent the irradiation of various organs with large vessels, and due to different tumor locations, not every spleen or bone marrow of each patient was covered within the same scanning range. On the other hand, the bone marrow in this study was delineated by the external contours rather than the low-density regions within the bones.

There are some limitations in our study. First, it is a retrospective study, meaning that we could hardly control other factors that may affect ALCs, such as different chemotherapy regimens. Second, flow cytometry was not applied to identify the lymphocyte diversity, which plays a prominent part in immune response. Third, this preliminary finding should be verified in a larger population. Fourth, the relative volumes of organs at risk utilized in this study may be susceptible to variation depending on factors such as the scan range and patient positioning. Fifth, newer edition for evaluating tumor stage should be applied in future research. Last, it's worth investigating which part of bone marrow takes more responsibility to lymphocyte recovery in esophageal cancer, or evaluating longitudinal changes in hematopoietic function of bone marrow by PET.

Despite these aforementioned limitations, our study yielded valuable insights into the association between an insufficient level of lymphocyte recovery after dCCRT and poorer survival outcomes in patients with ESCC, regardless of the severity of ALC nadir (G4 or G1–3) during radiotherapy. What’s more, our findings highlighted the potential impact of bone marrow irradiation on lymphocyte recovery, emphasizing the need for strict restrictions on nonessential bone marrow irradiation during radiotherapy..

## Conclusion

Inadequate lymphocyte recovery at 6 months after dCCRT for ESCC was an independent prognostic indicator of unfavorable survival outcomes, regardless of whether it occurred in patients who experienced G4 or G1–3 ALC nadir during radiotherapy. Additionally, there was a significant association between bone marrow irradiation and lymphocyte recovery.

### Supplementary Information

**Additional file 1:** Supplementary Figures 1–3.

**Additional file 2:** Supplementary Tables 1–8.

## Data Availability

Within the article and its additional files.

## Abbreviations

ALC: Absolute lymphocyte count
BM: Bone marrow
CI: Confidence interval
CRT: Chemoradiotherapy
dCCRT: Definitive concurrent chemoradiotherapy
DMFS: Distant metastasis-free survival
ECOG-PS: Eastern Cooperative Oncology Group performance status
EDIC: The effective dose to immune cells
ESCC: Esophageal squamous cell carcinoma
HR: Hazard ratio
LRFS: Local recurrence-free survival
LRI: Lymphocyte recovery index
OR: Odds ratio
OS: Overall survival
PET: Positron emission tomography
PF: Fluorouracil (5-FU) with cisplatin (DDP)
PFS: Progression-free survival
ROC: Receiver operating characteristic curve
TC: PTV: planning target volume
CBP: PTX with carboplatin
TF: 5-FU with paclitaxel (PTX)
TP: PTX with DDP
