# Supplementary material for: Lymphocyte recovery from radiation-induced lymphopenia in locally advanced esophageal squamous cell carcinoma: correlations with prognosis and lymphocyte-related organs
Source: Radiat Oncol. 2023 Oct 19;18:172. doi: 10.1186/s13014-023-02354-w (PMC10588237; doi:10.1186/s13014-023-02354-w)

**Supplement Figures**

Figure S1 The screening diagram of this study. *Abbreviations: ALC, absolute lymphocyte count; dCCRT, definitive concurrent chemoradiotherapy.*


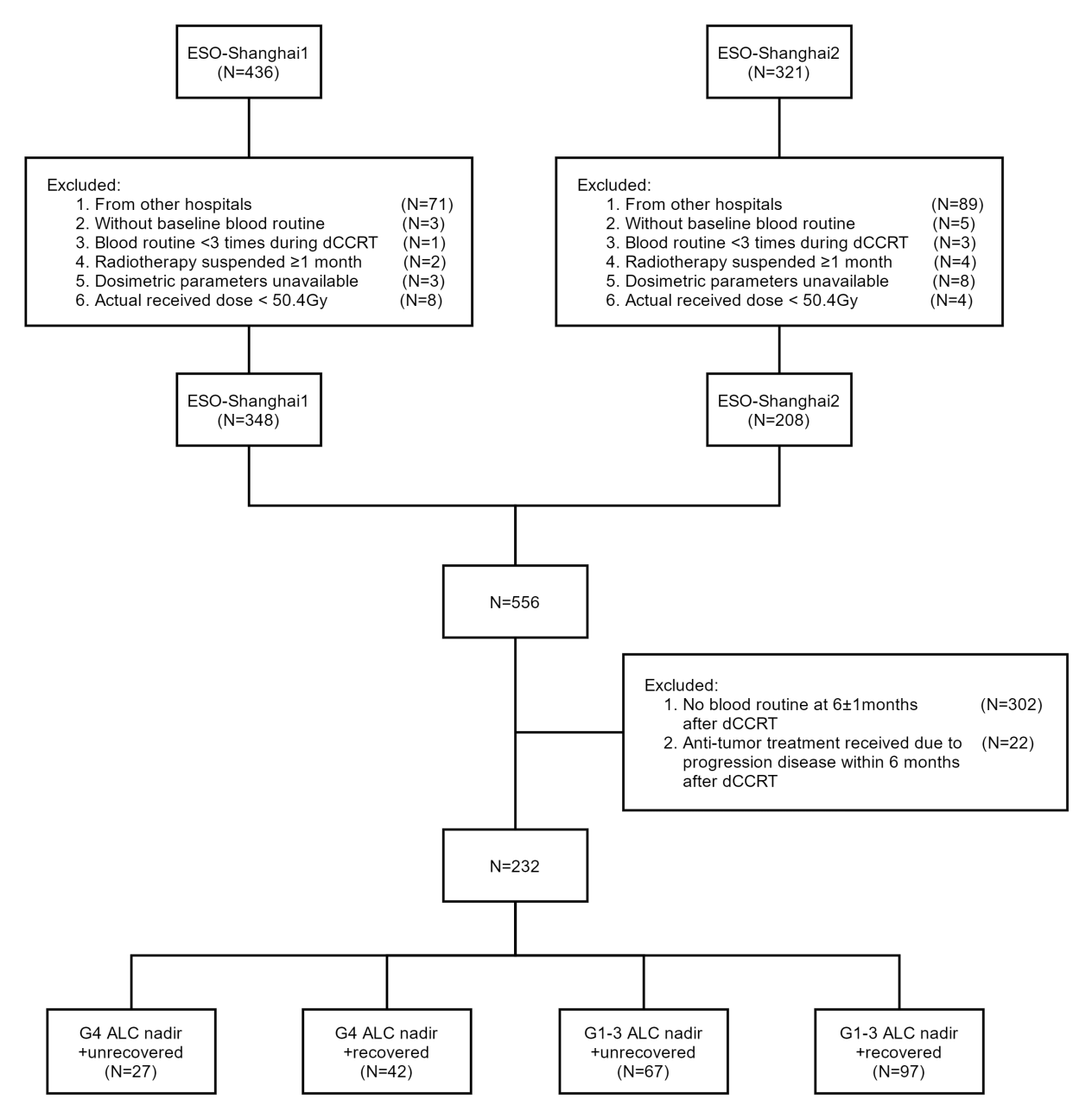


Figure S2 Maxstat analysis of lymphocyte recovery index (LRI) based on overall survival.


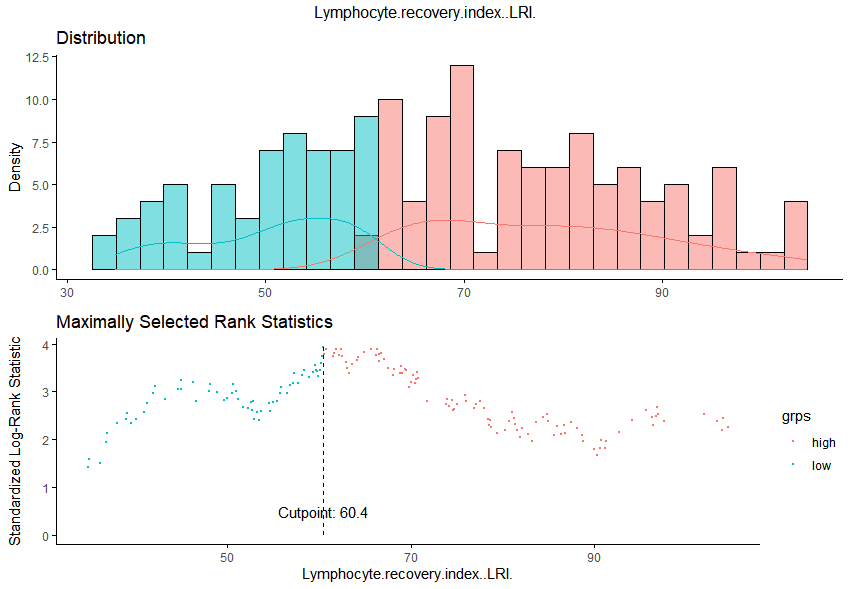


Figure S3 Kaplan Meier curves of (A) overall survival, (B) progression-free survival; (C) local recurrence*-free survival*, and (D) distant metastasis*-free survival* between groups of lymphocyte recovered (N=92) and unrecovered (N=82) in patients with stage III+IV. *Abbreviations: ALC, absolute lymphocyte count dCCRT, definitive concurrent chemoradiotherapy; DMFS, distant metastasis-free survival; LRFS, local recurrence-free survival; OS, overall survival; PFS, progression-free survival.*


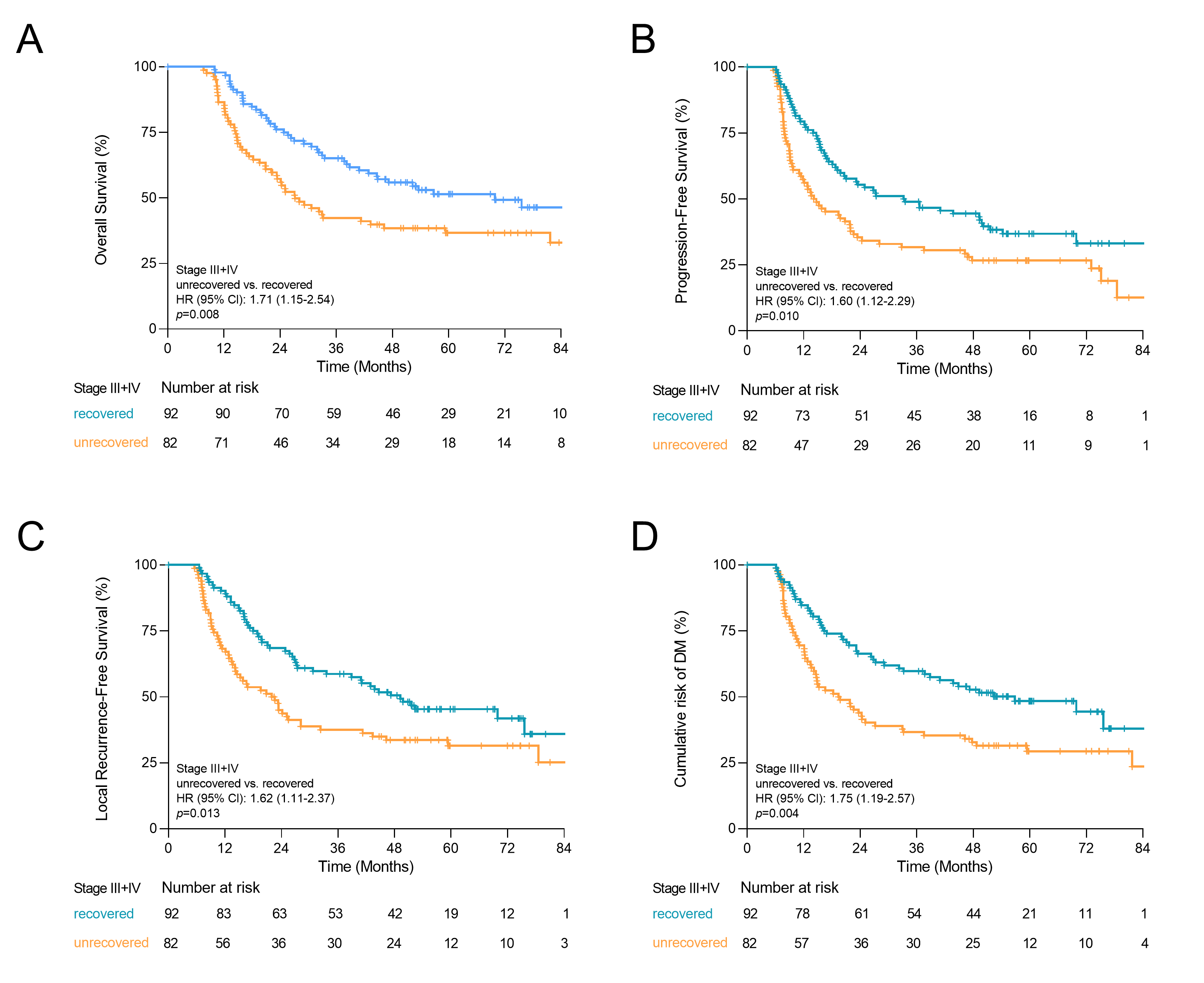

Supplement: Supplementary file 1 — Additional file 1: Supplementary Figures 1–3. [file 13014_2023_2354_MOESM1_ESM.docx]
