# Supplementary material for: Lymphocyte recovery from radiation-induced lymphopenia in locally advanced esophageal squamous cell carcinoma: correlations with prognosis and lymphocyte-related organs
Source: Radiat Oncol. 2023 Oct 19;18:172. doi: 10.1186/s13014-023-02354-w (PMC10588237; doi:10.1186/s13014-023-02354-w)
Supplement: Supplementary file 2 — Additional file 2: Supplementary Tables 1–8. [file 13014_2023_2354_MOESM2_ESM.docx]

***Supplement Tables***

Table S1 Distributions of patient characteristics in Group A, B, C, and D.

|  | Group A  (N=27) | Group B  (N=42) | Group C  (N=67) | Group D  (N=96) |
| --- | --- | --- | --- | --- |
| Gender |  |  |  |  |
| Male | 22 | 39 | 53 | 71 |
| Female | 5 | 3 | 14 | 25 |
| Age |  |  |  |  |
| ≤ 62y | 15 | 18 | 38 | 56 |
| > 62y | 12 | 24 | 29 | 40 |
| ECOG-PS |  |  |  |  |
| 0 | 18 | 24 | 51 | 69 |
| 1-2 | 9 | 18 | 16 | 27 |
| Tumor Stage ^*^ |  |  |  |  |
| II | 1 | 13 | 10 | 34 |
| III+IV | 26 | 29 | 57 | 62 |
| Tumor Location |  |  |  |  |
| Cervical+Upper | 13 | 17 | 46 | 61 |
| Middle+Lower+Multiple | 14 | 25 | 21 | 35 |
| Length |  |  |  |  |
| ≤ 5.0cm | 15 | 24 | 28 | 51 |
| > 5.0cm | 12 | 18 | 39 | 45 |
| Radiotherapy Dose |  |  |  |  |
| 61.2Gy | 25 | 39 | 67 | 89 |
| 50.4~ <61.2Gy | 2 | 3 | 0 | 7 |
| Chemo Regimen |  |  |  |  |
| PF | 2 | 7 | 28 | 27 |
| TF | 12 | 19 | 26 | 45 |
| TP | 6 | 10 | 6 | 8 |
| TC | 7 | 6 | 7 | 16 |
| Concurrent Chemo  Completion |  |  |  |  |
| Yes | 23 | 34 | 58 | 82 |
| No | 4 | 8 | 9 | 14 |
| Consolidation Chemo  Cycles |  |  |  |  |
| 2 cycles | 22 | 25 | 13 | 77 |
| 0-1 cycle | 5 | 17 | 54 | 19 |

*According to AJCC 6^th^. Group A included patients with G4 ALC nadir during dCCRT and LRI <60% (N=27); group B with G4 ALC nadir during dCCRT and LRI ≥60% (N=42); group C included patients with G1-3 ALC nadir during dCCRT and LRI <60% (N=67) and group D with G1-3 ALC nadir during dCCRT and LRI ≥60% (N=96). *Abbreviations: ALC: absolute lymphocyte count; ECOG-PS: Eastern Cooperative Oncology Group performance status; LRI, lymphocyte recovery index; PF: fluorouracil (5-FU) with cisplatin (DDP); TC: PTX with carboplatin (CBP); TF: 5-FU with paclitaxel (PTX); TP: PTX with DDP.*

Table S2 Univariable Cox analysis for survival outcomes between group A and group B.

|  | OS | |  | PFS | | |  | LRFS | | |  | | DMFS | |
| --- | --- | --- | --- | --- | --- | --- | --- | --- | --- | --- | --- | --- | --- | --- |
|  | HR (95% Cl) | *p* |  | HR (95% Cl) | *p* |  | | HR (95% Cl) | *p* |  | | HR (95% Cl) | | *p* |
| Gender |  | 0.190 |  |  | 0.236 |  | |  | 0.775 |  | |  | | 0.223 |
| Male | 2.19 (0.68-7.10) |  |  | 1.75 (0.70-4.39) |  |  | | 1.11 (0.54-2.31) |  |  | | 1.61 (0.75-3.45) | |  |
| Female | Ref |  |  | Ref |  |  | | Ref |  |  | | Ref | |  |
| Age |  | 0.339 |  |  | 0.556 |  | |  | 0.401 |  | |  | | 0.389 |
| ≤ 62y | 1.34 (0.73-2.47) |  |  | 1.17 (0.69-2.01) |  |  | | 1.28 (0.72-2.29) |  |  | | 1.29 (0.73-2.28) | |  |
| > 62y | Ref |  |  | Ref |  |  | | Ref |  |  | | Ref | |  |
| ECOG-PS |  | 0.660 |  |  | 0.894 |  | |  | 0.609 |  | |  | | 0.301 |
| 0 | 1.15 (0.61-2.16) |  |  | 1.04 (0.61-1.79) |  |  | | 1.17 (0.64-2.14) |  |  | | 0.73 (0.40-1.33) | |  |
| 1-2 | Ref |  |  | Ref |  |  | | Ref |  |  | | Ref | |  |
| Tumor Stage^*^ |  | 0.081 |  |  | 0.141 |  | |  | 0.055 |  | |  | | 0.066 |
| II | 0.46 (0.19-1.10) |  |  | 0.60 (0.30-1.19) |  |  | | 0.43 (0.18-1.02) |  |  | | 0.47 (0.21-1.05) | |  |
| III+IV | Ref |  |  | Ref |  |  | | Ref |  |  | | Ref | |  |
| Tumor Location |  | 0.554 |  |  | 0.821 |  | |  | 0.807 |  | |  | | 0.867 |
| Cervical+Upper | 1.20 (0.65-2.22) |  |  | 1.06 (0.62-1.82) |  |  | | 1.08 (0.60-1.93) |  |  | | 1.05 (0.59-1.88) | |  |
| Middle+Lower+Multiple | Ref |  |  | Ref |  |  | | Ref |  |  | | Ref | |  |
| Length |  | 0.188 |  |  | 0.448 |  | |  | 0.258 |  | |  | | 0.108 |
| ≤ 5.0cm | 0.66 (0.36-1.22) |  |  | 0.81 (0.47-1.39) |  |  | | 0.72 (0.40-1.28) |  |  | | 0.62 (0.35-1.11) | |  |
| > 5.0cm | Ref |  |  | Ref |  |  | | Ref |  |  | | Ref | |  |
| Radiotherapy Dose |  | 0.545 |  |  | 0.163 |  | |  | 0.432 |  | |  | | 0.441 |
| 61.2Gy | 1.44 (0.44-4.66) |  |  | 2.30 (0.72-7.38) |  |  | | 1.60 (0.50-5.17) |  |  | | 1.59 (0.50-5.12) | |  |
| 50.4~<61.2Gy | Ref |  |  | Ref |  |  | | Ref |  |  | | Ref | |  |
| Chemo Regimens |  | 0.689 |  |  | 0.790 |  | |  | 0.679 |  | |  | | 0.417 |
| PF | 0.77 (0.27-2.23) | 0.633 |  | 0.64 (0.24-1.69) | 0.368 |  | | 0.75 (0.27-2.12) | 0.593 |  | | 0.55 (0.20-1.52) | | 0.247 |
| TF | 1.01 (0.44-2.30) | 0.985 |  | 0.96 (0.46-2.01) | 0.915 |  | | 1.09 (0.50-2.37) | 0.838 |  | | 0.70 (0.33-1.49) | | 0.354 |
| TP | 0.63 (0.24-1.69) | 0.359 |  | 0.86 (0.37-1.99) | 0.717 |  | | 0.70 (0.28-1.77) | 0.454 |  | | 0.48 (0.20-1.19) | | 0.112 |
| TC | Ref |  |  | Ref |  |  | | Ref |  |  | | Ref | |  |
| Consolidation Chemo Cycles |  | 0.139 |  |  | 0.796 |  | |  | 0.296 |  | |  | | 0.719 |
| 2 cycles | 1.69 (0.84-3.36) |  |  | 0.93 (0.53-1.63) |  |  | | 1.19 (0.86-1.64) |  |  | | 1.54 (0.72-3.30) | |  |
| 0 -1cycle | Ref |  |  | Ref |  |  | | Ref |  |  | | Ref | |  |
| Groups |  | **<0.001** |  |  | **<0.001** |  | |  | **<0.001** |  | |  | | **<0.001** |
| Group A | 3.10 (1.67-5.73) |  |  | 3.67 (2.09-6.46) |  |  | | 3.17 (1.75-5.75) |  |  | | 3.96 (2.18-7.21) | |  |
| Group B | Ref |  |  | Ref |  |  | | Ref |  |  | | Ref | |  |

*According to AJCC 6^th^. Group A included patients with G4 ALC nadir during dCCRT and LRI <60% (N=27) and group B with G4 ALC nadir during dCCRT and LRI ≥60% (N=42). *Abbreviations: ALC: absolute lymphocyte count; CI, confidence interval; DMFS, distant metastasis-free survival; ECOG-PS: Eastern Cooperative Oncology Group performance status; HR: hazard ratio; LRFS, local recurrence-free survival; LRI, lymphocyte recovery index; OS, overall survival; PF: fluorouracil (5-FU) with cisplatin (DDP); PFS, progression-free survival; TC: PTX with carboplatin (CBP); TF: 5-FU with paclitaxel (PTX); TP: PTX with DDP..*

Table S3 Univariable Cox analysis for survival outcomes between group C and group D.

|  | OS | |  | | PFS | |  | LRFS | |  | DMFS | |
| --- | --- | --- | --- | --- | --- | --- | --- | --- | --- | --- | --- | --- |
|  | HR (95% Cl) | *p* |  | HR (95% Cl) | | *p* |  | HR (95% Cl) | *p* |  | HR (95% Cl) | *p* |
| Gender |  | 0.815 |  |  | | 0.246 |  |  | 0.634 |  |  | 0.402 |
| Male | 1.07 (0.62-1.83) |  |  | 1.36 (0.81-2.27) | |  |  | 1.13 (0.69-1.83) |  |  | 1.24 (0.75-2.05) |  |
| Female | Ref |  |  | Ref | |  |  | Ref |  |  | Ref |  |
| Age |  | 0.266 |  |  | | 0.110 |  |  | 0.310 |  |  | 0.295 |
| ≤ 62y | 1.30 (0.82-2.08) |  |  | 1.41 (0.92-2.17) | |  |  | 1.26 (0.81-1.98) |  |  | 1.28 (0.81-2.01) |  |
| > 62y | Ref |  |  | Ref | |  |  | Ref |  |  | Ref |  |
| ECOG-PS |  | 0.543 |  |  | | 0.566 |  |  | 0.469 |  |  | 0.541 |
| 0 | 1.18 (0.67-2.04) |  |  | 1.15 (0.71-1.86) | |  |  | 1.21 (0.72-2.03) |  |  | 1.18 (0.70-2.00) |  |
| 1-2 | Ref |  |  | Ref | |  |  | Ref |  |  | Ref |  |
| Tumor Stage^*^ |  | **0.005** |  |  | | **0.001** |  |  | **0.004** |  |  | **0.002** |
| II | 0.42 (0.22-0.77) |  |  | 0.40 (0.21-0.65) | |  |  | 0.42 (0.23-0.76) |  |  | 0.38 (0.20-0.70) |  |
| III+IV | Ref |  |  | Ref | |  |  | Ref |  |  | Ref |  |
| Tumor Location |  | **0.019** |  |  | | **0.007** |  |  | **0.015** |  |  | **0.009** |
| Cervical+Upper | 0.58 (0.37-0.92) |  |  | 0.56 (0.37-0.86) | |  |  | 0.58 (0.37-0.90) |  |  | 0.55 (0.35-0.86) |  |
| Middle+Lower+Multiple | Ref |  |  | Ref | |  |  | Ref |  |  | Ref |  |
| Length |  | **0.034** |  |  | | **0.019** |  |  | 0.057 |  |  | **0.013** |
| ≤ 5.0cm | 0.60 (0.39-1.00) |  |  | 0.60 (0.41-0.96) | |  |  | 0.65 (0.41-1.01) |  |  | 0.56 (0.35-0.88) |  |
| > 5.0cm | Ref |  |  | Ref | |  |  | Ref |  |  | Ref |  |
| Radiotherapy Dose |  | 0.394 |  |  | | 0.480 |  |  | 0.606 |  |  | 0.401 |
| 61.2Gy | 1.84 (0.45-7.51) |  |  | 1.52 (0.48-4.79) | |  |  | 1.36 (0.43-4.30) |  |  | 1.83 (0.45-7.43) |  |
| 50.4~<61.2Gy | Ref |  |  | Ref | |  |  | Ref |  |  | Ref |  |
| Chemo Regimens |  | 0.607 |  |  | | 0.588 |  |  | 0.714 |  |  | 0.495 |
| PF | 1.52 (0.69-3.32) | 0.297 |  | 1.09 (0.57-2.07) | | 0.795 |  | 1.04 (0.53-2.04) | 0.904 |  | 1.17 (0.59-2.34) | 0.653 |
| TF | 1.22 (0.56-2.64) | 0.623 |  | 0.87 (0.46-1.64) | | 0.666 |  | 0.81 (0.42-1.58) | 0.543 |  | 0.86 (0.43-1.70) | 0.655 |
| TP | 0.96 (0.31-2.93) | 0.938 |  | 0.64 (0.24-1.67) | | 0.357 |  | 0.73 (0.27-1.94) | 0.522 |  | 0.65 (0.23-1.88) | 0.431 |
| TC | Ref |  |  | Ref | |  |  | Ref |  |  | Ref |  |
| Consolidation Chemo Cycles |  | 0.758 |  |  | | 0.983 |  |  | 0.846 |  |  | 0.962 |
| 2 cycles | 1.10 (0.61-1.96) |  |  | 1.00 (0.60-1.65) | |  |  | 0.97 (0.75-1.27) |  |  | 1.01 (0.77-1.33) |  |
| 0=1 cycle | Ref |  |  | Ref | |  |  | Ref |  |  | Ref |  |
| Groups |  | **0.005** |  |  | | **0.017** |  |  | **0.009** |  |  | **0.008** |
| Group C | 1.91 (1.21-3.00) |  |  | 1.65 (1.10-2.50) | |  |  | 1.80 (1.16-2.80) |  |  | 1.82 (1.17-2.83) |  |
| Group D | Ref |  |  | Ref | |  |  | Ref |  |  | Ref |  |

*According to AJCC 6^th^. Group C included patients with G1-3 ALC nadir during dCCRT and LRI <60% (N=67) and group D with G1-3 ALC nadir during dCCRT and LRI ≥60% (N=96). *Abbreviations: ALC: absolute lymphocyte count; CI, confidence interval; DMFS, distant metastasis-free survival; ECOG-PS: Eastern Cooperative Oncology Group performance status; HR: hazard ratio; LRFS, local recurrence-free survival; LRI, lymphocyte recovery index; OS, overall survival; PF: fluorouracil (5-FU) with cisplatin (DDP); PFS, progression-free survival; TC: PTX with carboplatin (CBP); TF: 5-FU with paclitaxel (PTX); TP: PTX with DDP.*

Table S4 Multivariate Cox analysis for survival outcomes between group C and D.

|  | OS | |  | PFS | |  | LRFS | |  | DMFS | |
| --- | --- | --- | --- | --- | --- | --- | --- | --- | --- | --- | --- |
|  | HR (95% Cl) | *p* |  | HR (95% Cl) | *p* |  | HR (95% Cl) | *p* |  | HR (95% Cl) | *p* |
| Tumor Stage^*^ |  | **0.014** |  |  | **0.001** |  |  | **0.009** |  |  | **0.004** |
| II | 0.43 (0.22-0.84) |  |  | 0.35 (0.18-0.65) |  |  | 0.42 (0.22-0.81) |  |  | 0.38 (0.19-0.73) |  |
| III+IV | Ref |  |  | Ref |  |  | Ref |  |  | Ref |  |
| Tumor Location |  | **0.002** |  |  | **<0.001** |  |  | **0.002** |  |  | **0.001** |
| Cervical+Upper | 0.47 (0.29-0.76) |  |  | 0.45 (0.30-0.70) |  |  | 0.48 (0.31-0.76) |  |  | 0.44 (0.28-0.70) |  |
| Middle+Lower+Multiple | Ref |  |  | Ref |  |  | Ref |  |  | Ref |  |
| Length |  | 0.459 |  |  | 0.492 |  |  | 0.663 |  |  | 0.375 |
| ≤ 5.0cm | 0.83 (0.51-1.36) |  |  | 0.86 (0.55-1.33) |  |  | 0.90 (0.56-1.44) |  |  | 0.78 (0.49-1.26) |  |
| > 5.0cm | Ref |  |  | Ref |  |  | Ref |  |  | Ref |  |
| Groups |  | **0.025** |  |  | 0.121 |  |  | **0.040** |  |  | 0.060 |
| Group C | 1.70 (1.07-2.72) |  |  | 1.40 (0.92-2.13) |  |  | 1.60 (1.01-2.52) |  |  | 1.55 (0.98-2.44) |  |
| Group D | Ref |  |  | Ref |  |  | Ref |  |  | Ref |  |

*According to AJCC 6^th^. Group A included patients with G4 ALC nadir during dCCRT and LRI <60% (N=27) and group B with G4 ALC nadir during dCCRT and LRI ≥60% (N=42). *Abbreviations: ALC: absolute lymphocyte count; CI, confidence interval; DMFS, distant metastasis-free survival; ECOG-PS: Eastern Cooperative Oncology Group performance status; HR: hazard ratio; LRFS, local recurrence-free survival; LRI, lymphocyte recovery index; OS, overall survival; PF: fluorouracil (5-FU) with cisplatin (DDP); PFS, progression-free survival; TC: PTX with carboplatin (CBP); TF: 5-FU with paclitaxel (PTX); TP: PTX with DDP.*

Table S5 Univariable Cox analysis for survival outcomes between lymphocyte recovered and unrecovered within stage III+IV.

|  | OS | |  | PFS | | |  | LRFS | |  | | DMFS | |
| --- | --- | --- | --- | --- | --- | --- | --- | --- | --- | --- | --- | --- | --- |
|  | HR (95% Cl) | *p* |  | HR (95% Cl) | *p* |  | | HR (95% Cl) | *p* |  | HR (95% Cl) | | *p* |
| Gender |  | 0.048 |  |  | 0.156 |  | |  | 0.201 |  |  | | 0.060 |
| Male | 1.66 (1.00-2.73) |  |  | 1.36 (0.89-2.07) |  |  | | 1.35 (0.85-2.12) |  |  | 1.57 (0.98-2.51) | |  |
| Female | Ref |  |  | Ref |  |  | | Ref |  |  | Ref | |  |
| Age |  | 0.170 |  |  | 0.110 |  | |  | 0.209 |  |  | | 0.365 |
| ≤ 62y | 1.22 (0.89-1.99) |  |  | 1.35 (0.94-1.94) |  |  | | 1.28 (0.87-1.89) |  |  | 1.20 (0.81-1.76) | |  |
| > 62y | Ref |  |  | Ref |  |  | | Ref |  |  | Ref | |  |
| ECOG-PS |  | 0.669 |  |  | 0.813 |  | |  | 0.430 |  |  | | 0.516 |
| 0 | 1.10 (0.71-1.67) |  |  | 1.05 (0.71-1.54) |  |  | | 1.20 (0.79-1.82) |  |  | 1.15 (0.76-1.75) | |  |
| 1-2 | Ref |  |  | Ref |  |  | | Ref |  |  | Ref | |  |
| Tumor Location |  | **0.010** |  |  | **0.005** |  | |  | **0.011** |  |  | | **0.009** |
| Cervical+Upper | 0.57 (0.37-0.88) |  |  | 0.60 (0.42-0.86) |  |  | | 0.58 (0.38-0.88) |  |  | 0.60 (0.41-0.88) | |  |
| Middle+Lower+Multiple | Ref |  |  | Ref |  |  | | Ref |  |  | Ref | |  |
| Length |  | 0.131 |  |  | 0.194 |  | |  | 0.833 |  |  | | 0.143 |
| ≤ 5.0cm | 0.72 (0.46-1.11) |  |  | 0.77 (0.52-1.14) |  |  | | 0.95 (0.56-1.61) |  |  | 0.68 (0.41-.114) | |  |
| > 5.0cm | Ref |  |  | Ref |  |  | | Ref |  |  | Ref | |  |
| Radiotherapy Dose |  | 0.717 |  |  | 0.424 |  | |  | 0.531 |  |  | | 0.728 |
| 61.2Gy | 1.20 (0.44-3.28) |  |  | 1.50 (0.55-4.07) |  |  | | 1.38 (0.51-3.74) |  |  | 1.19 (0.44-3.25) | |  |
| 50.4~<61.2Gy | Ref |  |  | Ref |  |  | | Ref |  |  | Ref | |  |
| Chemo Regimens |  | 0.830 |  |  | 0.961 |  | |  | 0.895 |  |  | | 0.560 |
| PF | 1.09 (0.58-2.06) | 0.783 |  | 0.87 (0.50-1.52) | 0.633 |  | | 0.93 (0.52-1.67) | 0.803 |  | 0.80 (0.45-1.41) | | 0.438 |
| TF | 1.09 (0.61-1.97) | 0.767 |  | 0.88 (0.53-1.47) | 0.634 |  | | 0.97 (0.57-1.65) | 0.909 |  | 0.75 (0.44-1.27) | | 0.277 |
| TP | 0.81 (0.37-1.76) | 0.594 |  | 0.87 (0.45-1.67) | 0.672 |  | | 0.78 (0.38-1.57) | 0.478 |  | 0.61 (0.30-1.25) | | 0.175 |
| TC | Ref |  |  | Ref |  |  | | Ref |  |  | Ref | |  |
| Consolidation Chemo Cycles |  | 0.135 |  |  | 0.592 |  | |  | 0.289 |  |  | | 0.568 |
| 2 cycles | 1.22 (0.94-1.59) |  |  | 1.06 (0.85-1.32) |  |  | | 1.14 (0.90-1.45) |  |  | 1.07 (0.85-1.36) | |  |
| 0 -1cycle | Ref |  |  | Ref |  |  | | Ref |  |  | Ref | |  |
| Groups |  | **0.008** |  |  | **0.010** |  | |  | **0.013** |  |  | | **0.004** |
| Recovered | 1.71 (1.15-2.54) |  |  | 1.60 (1.12-2.29) |  |  | | 1.62 (1.11-2.37) |  |  | 1.75 (1.19-2.57) | |  |
| Unrecovered | Ref |  |  | Ref |  |  | | Ref |  |  | Ref | |  |

*Abbreviations: ALC: absolute lymphocyte count; CI, confidence interval; DMFS, distant metastasis-free survival; ECOG-PS: Eastern Cooperative Oncology Group performance status; HR: hazard ratio; LRFS, local recurrence-free survival; LRI, lymphocyte recovery index; OS, overall survival; PF: fluorouracil (5-FU) with cisplatin (DDP); PFS, progression-free survival; TC: PTX with carboplatin (CBP); TF: 5-FU with paclitaxel (PTX); TP: PTX with DDP..*

Table S6 Multivariable Cox analysis for survival outcomes between lymphocyte recovered and unrecovered within stage III+IV.

|  | OS | |  | PFS | | |  | LRFS | |  | | DMFS | |
| --- | --- | --- | --- | --- | --- | --- | --- | --- | --- | --- | --- | --- | --- |
|  | HR (95% Cl) | *p* |  | HR (95% Cl) | *p* |  | | HR (95% Cl) | *p* |  | HR (95% Cl) | | *p* |
| Gender |  | **0.046** |  | —— | —— |  | | —— | —— |  |  | | 0.068 |
| Male | 1.67 (1.01-2.75) |  |  |  |  |  | |  |  |  | 1.55 (0.87-2.48) | |  |
| Female | Ref |  |  |  |  |  | |  |  |  | Ref | |  |
| Tumor Location |  | **0.032** |  |  | **0.004** |  | |  | 0.024 |  |  | | **0.005** |
| Cervical+Upper | 0.65 (0.43-0.96) |  |  | 0.58 (0.41-0.84) |  |  | | 0.64 (0.44-0.94) |  |  | 0.57 (0.39-0.84) | |  |
| Middle+Lower+Multiple | Ref |  |  | Ref |  |  | | Ref |  |  | Ref | |  |
| Groups |  | **0.005** |  |  | **0.007** |  | |  | **0.010** |  |  | | **0.003** |
| Recovered | 1.77 (1.19-2.64) |  |  | 1.64 (1.15-2.35) |  |  | | 1.65 (1.13-2.42) |  |  | 1.80 (1.23-2.65) | |  |
| Unrecovered | Ref |  |  | Ref |  |  | | Ref |  |  | Ref | |  |

*Abbreviations: ALC: absolute lymphocyte count; CI, confidence interval; DMFS, distant metastasis-free survival; ECOG-PS: Eastern Cooperative Oncology Group performance status; HR: hazard ratio; LRFS, local recurrence-free survival; LRI, lymphocyte recovery index; OS, overall survival; PF: fluorouracil (5-FU) with cisplatin (DDP); PFS, progression-free survival; TC: PTX with carboplatin (CBP); TF: 5-FU with paclitaxel (PTX); TP: PTX with DDP..*

Table S7 Univariable Logistic analysis for lymphocyte recovery among patients with G4 ALC nadir during dCCRT.

|  | | Univariable | |
| --- | --- | --- | --- |
|  | | OR (95% Cl) | *p* |
| Gender | |  | 0.163 |
| Male | | 0.34 (0.07-1.55) |  |
| Female | | Ref |  |
| Age | |  | 0.304 |
| ≤ 62y | | 1.67 (0.63-4.42) |  |
| > 62y | | Ref |  |
| ECOG-PS | |  | 0.430 |
| 0 | | 1.50 (0.55-4.11) |  |
| 1-2 | | Ref |  |
| Tumor Stage^*^ | |  | **0.022** |
| II | | 0.09 (0.01-0.70) |  |
| III+IV | | Ref |  |
| Tumor Location | |  | 0.531 |
| Cervical+Upper | | 1.37 (0.52-3.62) |  |
| Middle+Lower+Multiple | | Ref |  |
| Length | |  | 0.897 |
| ≤ 5.0cm | | 0.94 (0.35-2.48) |  |
| > 5.0cm | | Ref |  |
| Radiatherapy Dose | |  | 0.967 |
| 61.2Gy | | 0.96 (0.15-6.19) |  |
| 50.4~<61.2Gy | | Ref |  |
| Chemo Regimens | |  | 0.535 |
| PF | | 0.25 (0.04-1.66) | 0.149 |
| TF | | 0.54 (0.15-2.00) | 0.358 |
| TP | | 0.51 (0.12-2.28) | 0.381 |
| TC | | Ref |  |
| Consolidation Chemo Cycles | |  | 0.062 |
| 2 cycles | | 2.99 (0.95-9.45) |  |
| 0-1 cycle | | Ref |  |
| Bone Marrow, % | |  |  |
| V5 | ≥ 40.7 | 5.75 (1.69-19.52) | **0.005** |
|  | < 40.7 | Ref |  |
| V10 | ≥ 37.0 | 4.56 (1.56-13.46) | **0.006** |
|  | < 37.0 | Ref |  |
| V20 | ≥ 25.9 | 2.20 (0.81-6.00) | 0.124 |
|  | < 25.9 | Ref |  |
| V30 | ≥ 15.9 | 2.30 (0.66-8.07) | 0.193 |
|  | < 15.9 | Ref |  |
| V50 | ≥ 2.5 | 0.40 (0.06-2.57) | 0.334 |
|  | < 2.5 | Ref |  |
| Spleen^§^ | |  | NS |
| EDIC | ≥ 11.8Gy | 0.57 (0.19-1.74) | 0.324 |
|  | < 11.8Gy | Ref |  |

*According to AJCC 6^th^. §No specific cut-off values were obtained in the spleen V5, V10, V20, V30 and V50 by receiver operating characteristic curve (ROC) analysis.

*Abbreviations: ALC: absolute lymphocyte count; CI, confidence interval; dCCRT, definitive concurrent chemoradiotherapy; ECOG-PS: Eastern Cooperative Oncology Group performance status; EDIC, effective dose to immune cells; OR, odds ratio; PF: fluorouracil (5-FU) with cisplatin (DDP); TC: PTX with carboplatin (CBP); TF: 5-FU with paclitaxel (PTX); TP: PTX with DDP.*

Table S8 Univariable Logistic analysis for lymphocyte recovery among patients with G1-3 ALC nadir during dCCRT.

|  | | Univariable | |
| --- | --- | --- | --- |
|  | | OR (95% Cl) | *p* |
| Gender | |  | 0.449 |
| Male | | 1.33 (0.63-2.81) |  |
| Female | | Ref |  |
| Age | |  | 0.837 |
| ≤ 62y | | 0.94 (0.50-1.76) |  |
| > 62y | | Ref |  |
| ECOG-PS | |  | 0.546 |
| 0 | | 1.25 (0.61-2.55) |  |
| 1-2 | | Ref |  |
| Tumor Stage^*^ | |  | **0.005** |
| II | | 0.32 (0.15-0.71) |  |
| III+IV | | Ref |  |
| Tumor Location | |  | 0.499 |
| Cervical+Upper | | 1.26 (0.65-2.44) |  |
| Middle+Lower+Multiple | | Ref |  |
| Length | |  | 0.155 |
| ≤ 5.0cm | | 0.63 (0.34-1.19) |  |
| > 5.0cm | | Ref |  |
| Radiatherapy Dose | |  | 0.999 |
| 61.2Gy | | — |  |
| 50.4~<61.2Gy | | Ref |  |
| Chemo Regimens | |  | 0.281 |
| PF | | 2.37 (0.84-6.66) | 0.102 |
| TF | | 1.32 (0.48-3.63) | 0.590 |
| TP | | 1.71 (0.43-6.83) | 0.445 |
| TC | | Ref |  |
| Consolidation Chemo Cycles | |  | 0.951 |
| 2 cycles | | 1.03 (0.47-2.25) |  |
| 0-1 cycle | | Ref |  |
| Bone Marrow, % | |  |  |
| V5 | ≥ 46.0 | 2.91 (1.50-5.66) | **0.002** |
|  | < 46.0 | Ref |  |
| V10 | ≥ 32.6 | 1.86 (0.99-3.52) | 0.054 |
|  | < 32.6 | Ref |  |
| V20 | ≥ 22.4 | 2.09 (1.04-4.18) | **0.038** |
|  | < 22.4 | Ref |  |
| V30 | ≥ 23.1 | 1.85 (0.90-3.80) | 0.094 |
|  | < 23.1 | Ref |  |
| V50 | ≥ 4.6 | 1.53 (0.80-2.92) | 0.204 |
|  | < 4.6 | Ref |  |
| Spleen, %^§^ | |  |  |
| V20 | ≥ 50.3 | 1.44 (0.09-23.42) | 0.798 |
|  | < 50.3 | Ref |  |
| V30 | ≥ 25.4 | 1.44 (0.09-23.42) | 0.798 |
|  | < 25.4 | Ref |  |
| V50 | ≥ 4.0 | 1.44 (0.09-23.42) | 0.798 |
|  | < 4.0 | Ref |  |
| EDIC | ≥ 10.3 Gy | 1.92 (0.98-3.74) | 0.056 |
|  | < 10. 3Gy | Ref |  |

*According to AJCC 6^th^. §No specific cut-off values were obtained in the spleen V5 and V10 by receiver operating characteristic curve (ROC) analysis.

*Abbreviations: ALC: absolute lymphocyte count; CI, confidence interval; dCCRT, definitive concurrent chemoradiotherapy; ECOG-PS: Eastern Cooperative Oncology Group performance status; EDIC, effective dose to immune cells; OR, odds ratio; PF: fluorouracil (5-FU) with cisplatin (DDP); TC: PTX with carboplatin (CBP); TF: 5-FU with paclitaxel (PTX); TP: PTX with DDP.*
